# Multi-Focus Image Fusion via Distance-Weighted Regional Energy and Structure Tensor in NSCT Domain

**DOI:** 10.3390/s23136135

**Published:** 2023-07-04

**Authors:** Ming Lv, Liangliang Li, Qingxin Jin, Zhenhong Jia, Liangfu Chen, Hongbing Ma

**Affiliations:** 1 College of Information Science and Engineering, Xinjiang University, Urumqi 830046, China; 2 School of Information and Electronics, Beijing Institute of Technology, Beijing 100081, China; 3 School of Mechanical Engineering, Guangxi University, Nanning 530004, China; 4 State Key Laboratory of Remote Sensing Science, Aerospace Information Research Institute, Chinese Academy of Sciences, Beijing 100101, China; 5 Department of Electronic Engineering, Tsinghua University, Beijing 100084, China

**Keywords:** multi-focus image, image fusion, distance-weighted regional energy, structure tensor, non-subsampled contourlet transform

## Abstract

In this paper, a multi-focus image fusion algorithm via the distance-weighted regional energy and structure tensor in non-subsampled contourlet transform domain is introduced. The distance-weighted regional energy-based fusion rule was used to deal with low-frequency components, and the structure tensor-based fusion rule was used to process high-frequency components; fused sub-bands were integrated with the inverse non-subsampled contourlet transform, and a fused multi-focus image was generated. We conducted a series of simulations and experiments on the multi-focus image public dataset Lytro; the experimental results of 20 sets of data show that our algorithm has significant advantages compared to advanced algorithms and that it can produce clearer and more informative multi-focus fusion images.

## 1. Introduction

**Figure 1:**
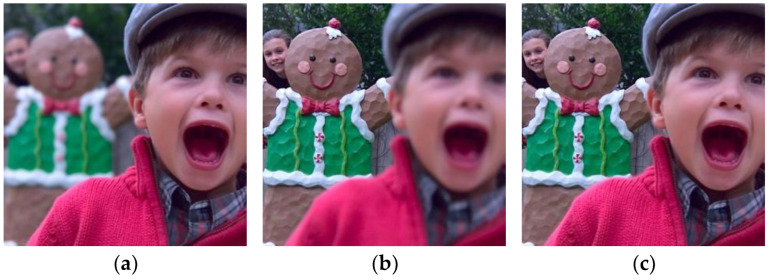
Multi-focus image fusion. (**a**) Near-focused image; (**b**) far-focused image; and (**c**) fused image.

In order to obtain richer and more useful information and to improve the completeness and accuracy of scene description, multi-focus images obtained from video sensors are usually fused [1,2]. Multi-focus image fusion uses effective information processing methods to fuse clear and focused information from different images, resulting in a high-definition fully focused image [3,4,5]. Figure 1 depicts an example of multi-focus image fusion.

After many years of extensive and in-depth research, the image fusion method has made significant progress. Image fusion approaches can be divided into three categories: transform domain-based, edge-preserving filtering-based, and deep learning-based algorithms [6,7,8,9].

The most widely used transform domain-based methods are the curvelet transform [10], contourlet transform [11,12], shearlet transform [13,14], etc. Kumar et al. [15] constructed an intelligent multi-modal image fusion technique utilizing the fast discrete curvelet transform and type-2 fuzzy entropy, and the fusion results demonstrate the efficiency of this model in terms of subjective and objective assessment. Kumar et al. [16] constructed an image fusion technique via an improved multi-objective meta-heuristic algorithm with fuzzy entropy in fast discrete curvelet transform domain; a comparison of the developed methodology over the state-of-the-art models observes enhanced performance with respect to visual quality assessment. Li et al. [17] integrated the curvelet and discrete wavelet transform for multi-focus image fusion, and it can obtain advanced fusion performance. Zhang et al. [18] constructed an image fusion model using the contourlet transform. The average-based fusion rule and region energy-based fusion rule are performed on the low- and high-frequency sub-bands, respectively; the fused image is analyzed using fast non-local clustering for multi-temporal synthetic aperture radar image change detection; and this technique generates state-of-the-art change detection performance on both small- and large-scale datasets. Li et al. [19] constructed an image fusion approach using sparse representation and local energy in shearlet transform domain; this method can obtain good fusion performance. Hao et al. [20] introduced a multi-scale decomposition optimization-based image fusion method via gradient-weighted local energy; the non-subsampled shearlet transform is utilized to decompose the source images into low- and high-frequency sub-images, and then the low-frequency components are divided into base layers and texture layers, the base layers are merged according to the intrinsic attribute-based energy fusion rule, and the structure tensor-based gradient-weighted local energy operator fusion rule is utilized to merge the texture layers and high-frequency sub-bands. This fusion method can achieve a superior fusion capability in both qualitative and quantitative assessments.

The methods based on edge-preserving filtering also hold an important position in the field of image fusion, such as guided image filtering [21], cross bilateral filtering [22], Gaussian curvature filtering [23], sub-window variance filtering [24], etc. Feng et al. [24] constructed the multi-channel dynamic threshold neural P systems-based image fusion method via a visual saliency map in the sub-window variance filter domain; this algorithm can obtain effective fusion performance both on visual quality and quantitative evaluations. Zhang et al. [25] introduced a double joint edge preservation filter-based image fusion technique via a non-globally saliency gradient operator; it can obtain excellent subjective and objective performance. Jiang et al. [26] introduced the image fusion approach utilizing entropy measure between intuitionistic fuzzy set joint Gaussian curvature filtering. 

The image fusion approaches based on deep learning have generated good performance, and these deep learning-based fusion models can be divided into supervised- and unsupervised-based approaches [27,28]. In terms of supervised algorithms, some effective fusion methods have been generated. The deep convolutional neural network introduced by Liu et al. [29] is used for multi-focus image fusion, and it generates good fusion results. The multi-scale visual attention deep convolutional neural network introduced by Lai et al. [30] is utilized to fuse multi-focus images. Wang et al. [31] introduced weakly supervised image fusion with modal synthesis and enhancement. The self-supervised residual feature learning model constructed by Wang et al. [32] is used to fuse multi-focus images. The attention mechanism-based image fusion approach via supervised learning was constructed by Jiang et al. [33]. In terms of unsupervised algorithms, Jin et al. [34] introduced the Transformer and U-Net for image fusion. Zhang et al. [35] presented another unsupervised generative adversarial network with adaptive and gradient joint constraints for image fusion. Liu et al. [36] constructed a generative adversarial network-based unsupervised back project dense network for image fusion. These deep learning-based fusion methods also achieve good performance.

In order to improve the fusion performance of multi-focus images, a distance-weighted regional energy-based multi-focus image fusion algorithm via the structure tensor in non-subsampled contourlet transform domain was constructed. The distance-weighted regional energy-based fusion rule and the structure tensor-based fusion rule were used to merge low- and high-frequency sub-images, respectively. The experimental results of the Lytro dataset show that the proposed fusion method can produce fusion effects that are superior to other traditional and deep learning methods in terms of subjective visual and objective evaluation.

## 2. Backgrounds

### 2.1. NSCT

**Figure 2:**
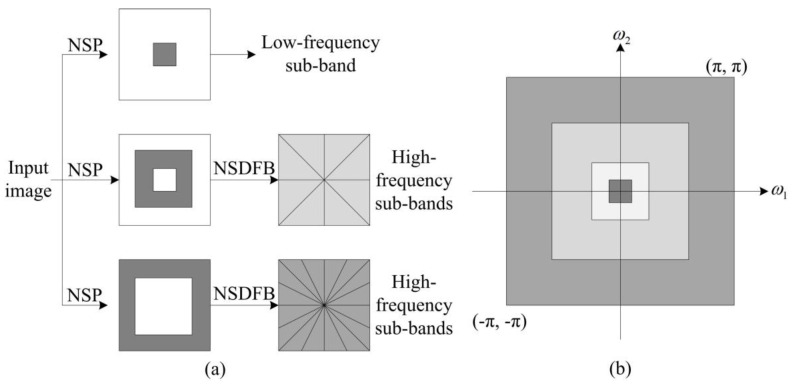
(**a**) NSCT basic framework structure diagram; (**b**) NSCT frequency domain partition diagram.

The non-subsampled contourlet transform (NSCT) is a transformation with multi-directional, multi-scale, and translation invariance [12]. Its basic framework structure is divided into two parts: the non-subsampled pyramid (NSP) decomposition mechanism and the non-subsampled directional filter bank (NSDFB) decomposition mechanism. The NSCT first utilizes NSP decomposition to perform multi-scale decomposition on the source image and then uses NSDFB decomposition to further decompose the high-frequency components in the direction, ultimately obtaining sub-band images of the source image at different scales and in different directions [12]. Figure 2 shows the NSCT basic framework structure diagram and the NSCT frequency domain partition diagram.

### 2.2. Structure Tensor

For a multi-channel image, $f\left(x,y\right)=\left(f_{1}\left(x,y\right),\;f_{2}\left(x,y\right),\ldots ,\;f_{3}\left(x,y\right)\right)$, the grayscale images and color images are two special cases when *m* = 1 and *m* = 3, respectively. The square of variation of $f\left(x,y\right)$ at position $\left(x,y\right)$ in direction θ for any $\varepsilon \to 0^{+}$ can be given by the following [37]:

(1) $$\begin{array}{l} {\left(df\right)}^{2}={\left\|f\left(x+\varepsilon \cos\theta ,y+\varepsilon \sin\theta \right)-f\left(x,y\right)\right\|}_{2}^{2}\\ \;\;\;\approx {\sum_{i=1}^{m}\left(\frac{\partial f_{i}}{\partial x}\varepsilon \cos\theta +\frac{\partial f_{i}}{\partial y}\varepsilon \sin\theta \right)}^{2} \end{array}$$

The rate of change $C\left(\theta \right)$ of image $f\left(x,y\right)$ at position $\left(x,y\right)$ can be expressed as follows:

(2) $$\begin{array}{l} C\left(\theta \right)=\sum_{i=1}^{m}{\left(\frac{\partial f_{i}}{\partial x}\cos\theta +\frac{\partial f_{i}}{\partial y}\sin\theta \right)}^{2}\\ \;\;=\left(\cos\theta ,\sin\theta \right)\left[\begin{array}{l} \sum_{i=1}^{m}{\left(\frac{\partial f_{i}}{\partial x}\right)}^{2}\sum_{i=1}^{m}\frac{\partial f_{i}}{\partial x}\frac{\partial f_{i}}{\partial y}\\ \sum_{i=1}^{m}\frac{\partial fi}{\partial y}\frac{\partial f_{i}}{\partial x}\sum_{i=1}^{m}{\left(\frac{\partial f_{i}}{\partial y}\right)}^{2} \end{array}\right]{\left(\cos\theta ,\sin\theta \right)}^{T}\\ \;\;=\left(\cos\theta ,\sin\theta \right)\sum_{i=1}^{m}\nabla f_{i}\nabla f_{i}{}^{T}{\left(\cos\theta ,\sin\theta \right)}^{T} \end{array}$$

where $\nabla f_{i}={\left(\frac{\partial f_{i}}{\partial x},\frac{\partial f_{i}}{\partial y}\right)}^{T}$, the following second-moment positive semi-definite matrix is

(3) $$P=\sum_{i=1}^{m}\nabla f_{i}\nabla f_{i}{}^{T}=\left[\begin{array}{l} EF\\ FG \end{array}\right]$$

where P is called the structure tensor and where *E*, *F*, and *G* are defined as

(4) $$E={\sum_{i=1}^{m}\left(\frac{\partial f_{i}}{\partial x}\right)}^{2}$$

(5) $$F=\sum_{i=1}^{m}\frac{\partial f_{i}}{\partial x}\frac{\partial f_{i}}{\partial y}$$

(6) $$G={\sum_{i=1}^{m}\left(\frac{\partial f_{i}}{\partial y}\right)}^{2}$$

The structure tensor-based focus detection operator (STO) is given by

(7) $$S=\sqrt{{\left(\lambda_{1}+\lambda_{2}\right)}^{2}+0.5{\left(\lambda_{1}-\lambda_{2}\right)}^{2}}$$

where $\lambda_{1}$ and $\lambda_{2}$ are the eigenvalues of the structure tensor and can be computed by the following:

(8) $$\lambda_{1,2}=\frac{1}{2}\left(\left(E+G\right)\pm \sqrt{{\left(G-E\right)}^{2}+4F^{2}}\right)$$

## 3. The Proposed Multi-Focus Image Fusion Method

**Figure 3:**
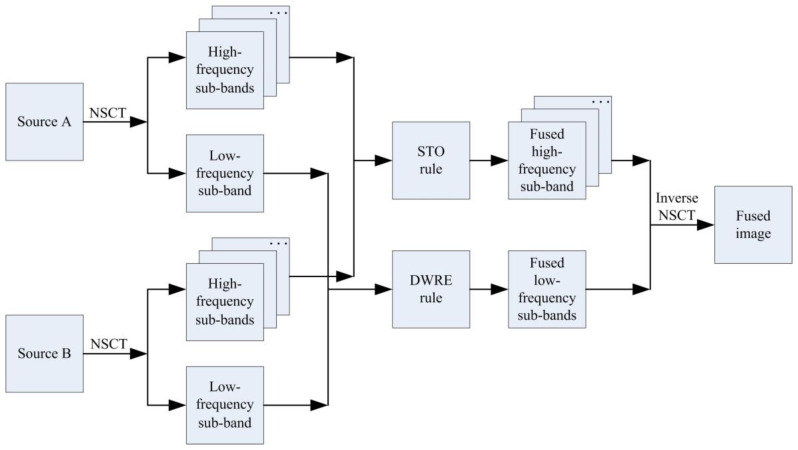
The schematic of the proposed fusion algorithm.

The multi-focus image fusion method based on the distance-weighted regional energy and structure tensor in NSCT domain is proposed in this section, and the schematic of the proposed algorithm is depicted in Figure 3. This fusion method can be divided into four parts: NSCT decomposition, low-frequency components fusion, high-frequency components fusion, and the inverse NSCT. More details can be seen in the follows.

### 3.1. NSCT Decomposition

The multi-focus images A and B were decomposed into low-frequency components and high-frequency components through the NSCT, and the corresponding coefficients are defined as $\left\{L_{A},H_{A}^{l,k}\right\}$ and $\left\{L_{B},H_{B}^{l,k}\right\}$, respectively. 

### 3.2. Low-Frequency Components Fusion

Low-frequency components contain more energy and information in the images. In this section, the distance-weighted regional energy (DWRE)-based rule was used to merge the low-pass sub-bands, and it is defined as follows [38]:

(9) $$DWRE^{L_{X}\left(i,j\right)}=\sum_{p=-1}^{1}\;\sum_{q=-1}^{1}W\left(p+2,q+2\right){\left(L_{X}\left(i+p,j+q\right)\right)}^{2}$$

where W shows a 3×3 matrix that allocates weights to the neighboring coefficients and where W is defined as

(10) $$W=\left[\begin{array}{ccc} \frac{1}{1+\sqrt{2}} & \frac{1}{2} & \frac{1}{1+\sqrt{2}}\\ \frac{1}{2} & 1 & \frac{1}{2}\\ \frac{1}{1+\sqrt{2}} & \frac{1}{2} & \frac{1}{1+\sqrt{2}} \end{array}\right]$$

Here, each entry of W is achieved by reciprocating ‘1 + distance of the respective position from its center, i.e., $W\left(2,2\right)$’, which is depicted in the following:

(11) $$W\left(i,j\right)=\frac{1}{1+\sqrt{{\left(i-2\right)}^{2}+{\left(j-2\right)}^{2}}}$$

The fused low-frequency component $L_{F}\left(i,j\right)$ is constructed by the following:

(12) $$L_{F}\left(i,j\right)=\left\{\begin{array}{l} L_{A}\left(i,j\right)\;\mathrm{if}\;DWRE^{L_{A}}\left(i,j\right)\geq DWRE^{L_{B}}\left(i,j\right)\\ L_{B}\left(i,j\right)\;\mathrm{else} \end{array}\right.$$

where $DWRE^{L_{X}}\left(i,j\right)|X\in \left\{A,B\right\}$ is the DWRE of $L_{X}\left(i,j\right)$ estimated over a 3×3 neighborhood centered at the $\left(i,j\right)$th coefficient.

### 3.3. High-Frequency Components Fusion

High-frequency components contain more details and noise in the images. In this section, the structure tensor-based focus detection operator (STO) fusion rule was used to process the high-frequency components, and the fused coefficients $H_{F}^{l,k}\left(i,j\right)$ can be computed by

(13) $$H_{F}^{l,k}\left(i,j\right)={\sum H}_{X}^{l,k}\left(i,j\right)\times {\tilde{M}}_{X}^{l,k}\left(i,j\right)X\in \left\{A,B\right\}$$

where

(14) $${\tilde{M}}_{X}^{l,k}\left(i,j\right)=\left\{\begin{array}{l} 1\;\mathrm{if}\;\sum_{\left(a,b\right)\in \Omega_{1}}\;M_{X}^{l,k}\left(i+a,j+b\right)>0.5\times size\left(\Omega_{1}\right)\\ 0\;\mathrm{else} \end{array}\right.$$

(15) $$M_{X}^{l,k}\left(i,j\right)=\left\{\begin{array}{l} 1\;\mathrm{if}\;\arg\max\left\{S_{A}^{l,k}\left(i,j\right),S_{B}^{l,k}\left(i,j\right)\right\}\\ 0\;\mathrm{else} \end{array}\right.$$

where $H_{X}^{l,k}\left(i,j\right)$ shows the high-frequency coefficient of image $X\in \left\{A,B\right\}$ at the l-th scale and k-th direction at location $\left(i,j\right)$ and where $S_{X}^{l,k}\left(i,j\right)$ shows the structure salient image of $H_{X}^{l,k}\left(i,j\right)$ computed by STO. $\Omega_{1}$ is a local region centered at $\left(i,j\right)$ with the size of $W_{1}\times V_{1}$. Equation (14) is the classical operator called the consistency verification, which can improve the robustness and rectify some wrong focusing detection of $M_{X}^{l,k}\left(i,j\right)$.

### 3.4. Inverse NSCT

The final fused image F was generated by the inverse NSCT, which was performed on fused low- and high-frequency components $\left\{L_{F},H_{F}^{l,k}\right\}$, and F is defined as follows:

(16) $$F\left(i,j\right)=L_{A}\left(i,j\right)+H_{F}^{l,k}\left(i,j\right)$$

The main steps of the proposed multi-focus image fusion approach are summarized in Algorithm 1.
**Algorithm 1** Proposed multi-focus image fusion method**Input:** the source images: A and B
**Parameters:** The number of NSCT decomposition levels: *L*, the number of directions at each decomposition level: K(l), l∈[1,L]
**Main step:**
**Step 1:** NSCT decomposition
For each source image X∈{A,B}
  Perform NSCT decomposition on
X to generate $\left\{L_{X},H_{X}^{l,k}\right\},\;l\in [1,L],\;k\in [1,K(l)]$;
End
**Step 2:** Low-frequency components fusion
For each source image
X∈{A,B}
  Calculate the DWRE for *L_X_* utilizing Equations (9)–(11);
End
Merge *L_A_* and *L_B_* utilizing Equations (12) to generate *L_F_*;
**Step 3:** High-frequency components fusion
For each Level
l=1:L
  For each direction
k=1:K(l)
   For each source image $X\in \left\{A,B\right\}$
    Calculate the structure salient image $S_{X}^{l,k}\left(i,j\right)$ of $H_{X}^{l,k}\left(i,j\right)$ computed by STO using Equation (7);
    Calculate the consistency verification using Equations (14) and (15);
   End
   Merge $H_{A}^{l,k}$ and $H_{B}^{l,k}$ using Equation (13)
  End
End
**Step 4.** Inverse NSCT
Perform inverse NSCT on
$\left\{L_{F},H_{F}^{l,k}\right\}$ to generate *F*;
**Output:** the fused image *F*. 

## 4. Experimental Results and Discussions

**Figure 4:**
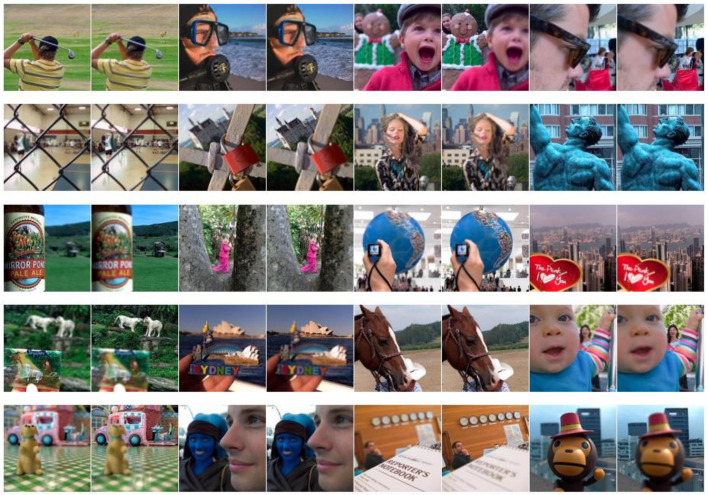
Lytro dataset.

In this section, the Lytro dataset with twenty pair images (Figure 4) are utilized to experiment, and eight state-of-the-art fusion algorithms are used to be compared with our method, namely, multi-focus image fusion using a bilateral-gradient-based sharpness criterion (BGSC) [39], the non-subsampled contourlet transform and fuzzy-adaptive reduced pulse-coupled neural network (NSCT) [40], multi-focus image fusion in gradient domain (GD) [41], the unified densely connected network for image fusion (FusionDN) [42], the fast unified image fusion network based on the proportional maintenance of gradient and intensity (PMGI) [43], image fusion based on target-enhanced multi-scale transform decomposition (TEMST) [44], the unified unsupervised image fusion network (U2Fusion) [45], and zero-shot multi-focus image fusion (ZMFF) [3]. Subjective and objective assessments are also used; in terms of objective assessment, eight metrics, including edge-based similarity measurement $Q_{AB/F}$ [46], the feature mutual information metric $Q_{FMI}$ [47], the gradient-based metric $Q_{G}$ [48], the structural similarity-based metric $Q_{E}$ [48], the phase congruency-based metric $Q_{P}$ [48], the structural similarity-based metric $Q_{Y}$ introduced by Yang et al. [48], the average gradient metric $Q_{AG}$ [22], and the average pixel intensity metric $Q_{API}$ [22], are used. The larger the values of these indicators, the better the fusion effect. In the proposed method, the size of $\Omega_{1}$ is set as 5 × 5; the ‘vk’ and ‘pyrexc’ are used as the pyramid filter and directional filter, respectively; and the four decomposition levels with 2, 2, 2, and 2 directions from a coarser scale to a finer scale are used.

### 4.1. Qualitative Comparisons

**Figure 5:**
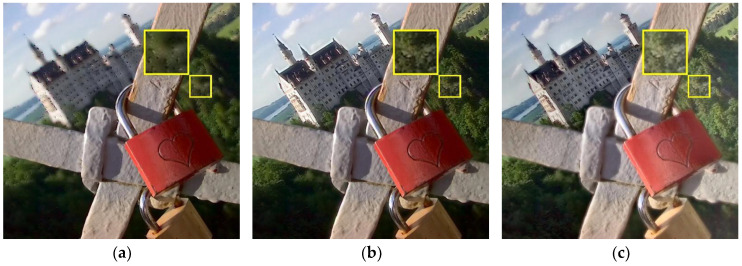

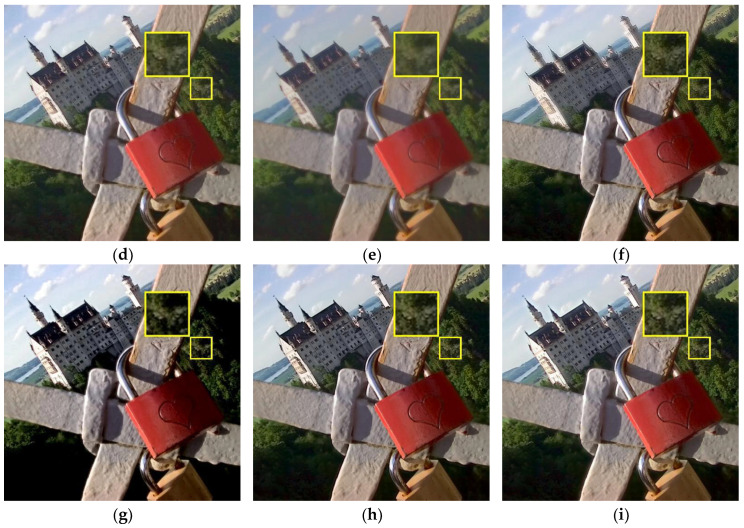
Lytro-06 fusion results. (**a**) BGSC; (**b**) NSCT; (**c**) GD; (**d**) FusionDN; (**e**) PMGI; (**f**) TEMST; (**g**) U2Fusion; (**h**) ZMFF; and (**i**) proposed.

**Figure 6:**
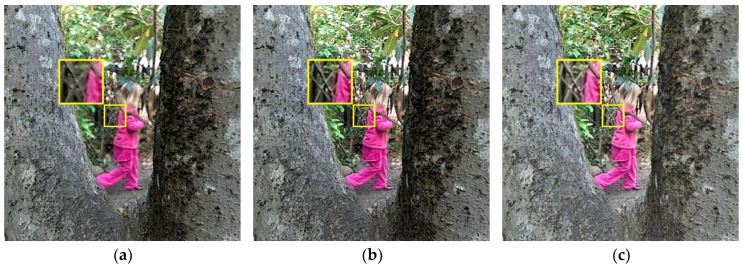

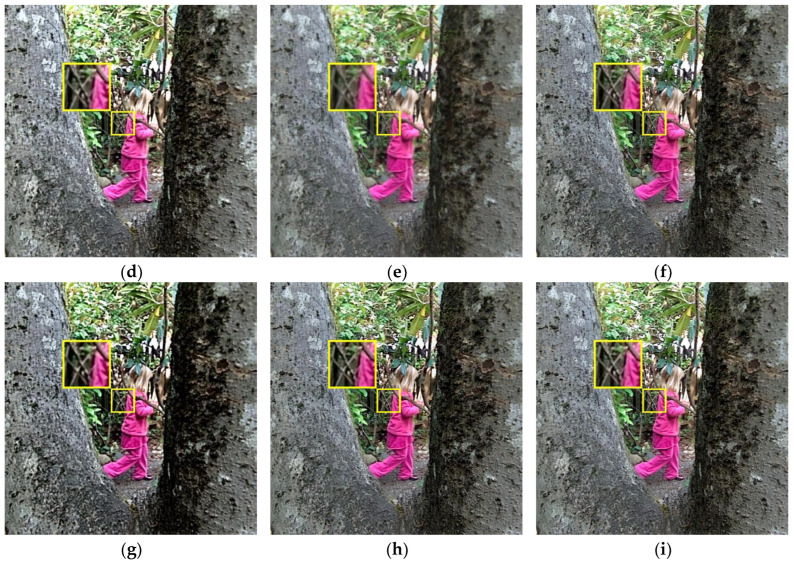
Lytro-10 fusion results. (**a**) BGSC; (**b**) NSCT; (**c**) GD; (**d**) FusionDN; (**e**) PMGI; (**f**) TEMST; (**g**) U2Fusion; (**h**) ZMFF; and (**i**) proposed.

**Figure 7:**
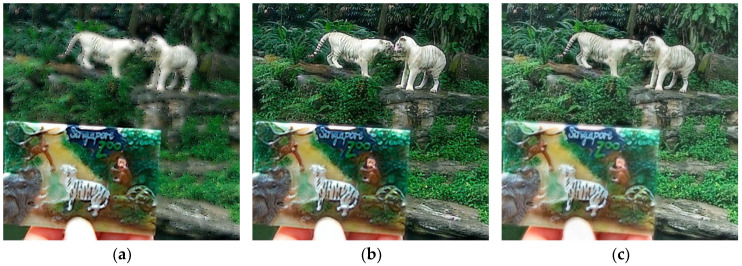

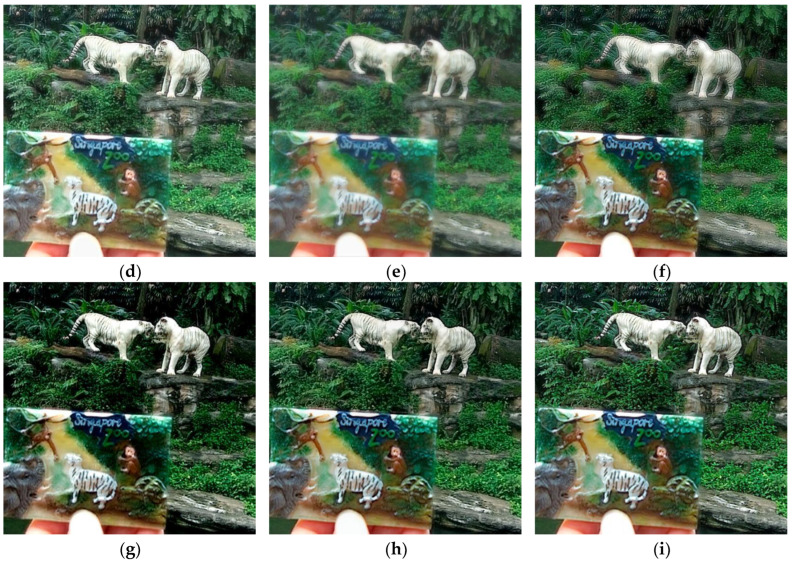
Lytro-13 fusion results. (**a**) BGSC; (**b**) NSCT; (**c**) GD; (**d**) FusionDN; (**e**) PMGI; (**f**) TEMST; (**g**) U2Fusion; (**h**) ZMFF; and (**i**) proposed.

**Figure 8:**
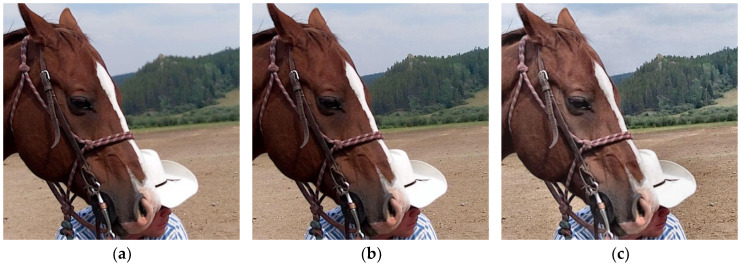

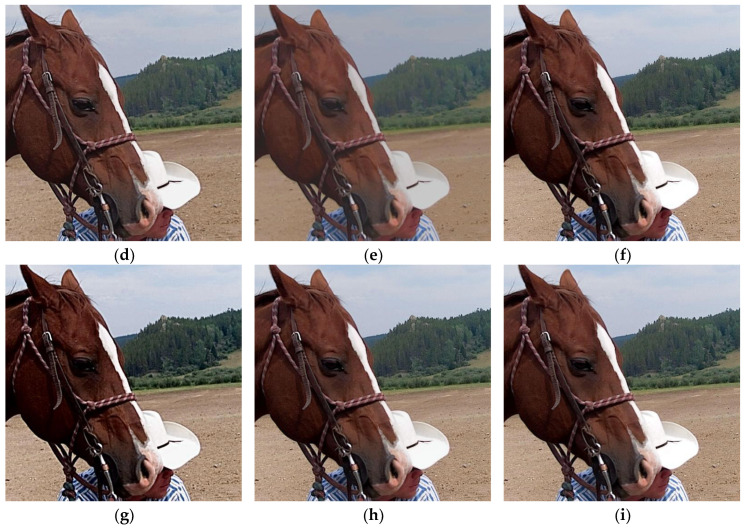
Lytro-15 fusion results. (**a**) BGSC; (**b**) NSCT; (**c**) GD; (**d**) FusionDN; (**e**) PMGI; (**f**) TEMST; (**g**) U2Fusion; (**h**) ZMFF; and (**i**) proposed.

**Figure 9:**
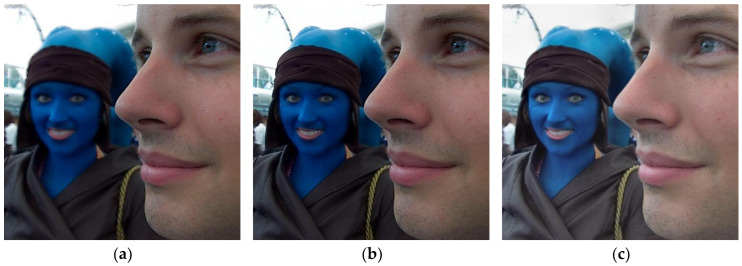

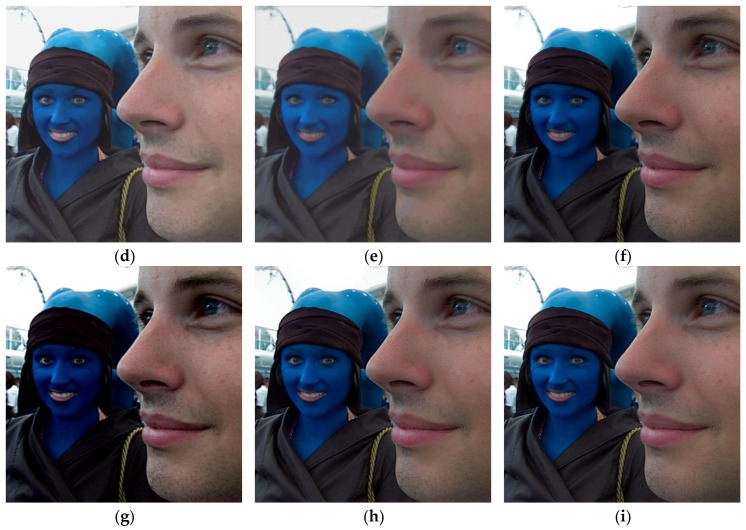
Lytro-18 fusion results. (**a**) BGSC; (**b**) NSCT; (**c**) GD; (**d**) FusionDN; (**e**) PMGI; (**f**) TEMST; (**g**) U2Fusion; (**h**) ZMFF; and (**i**) proposed.

In this section, we selected five sets of data from the Lytro dataset for result display; the qualitative comparisons of different methods are shown in Figure 5, Figure 6, Figure 7, Figure 8 and Figure 9. We have enlarged some areas for easy observation and comparison. In Figure 5, we can see that the fusion result generated by the BGSC method has a weak far-focus fusion effect and that black spots appear around the building; the fusion results computed by the NSCT, GD, FusionDN, PMGI, and TEMST methods are somewhat blurry; the U2Fusion method generates a dark fusion image which limits the observation of some areas in the image; and the ZMFF method produces fully focused image results. However, compared to our method, our result produces moderate brightness and clear edges, especially the brightness of the red lock in the image, which is better than ZMFF.

In Figure 6, we can see that the fusion results generated by the BGSC and PMGI methods are blurry, making it difficult to observe the detailed information in the fused images; the fusion images computed by NSCT, FusionDN, TEMST, and ZMFF are similar, these images all yielding fully focused image results; the GD method generates a high-brightness fusion image, with the color information in the image being partially lost; the U2Fusion method generates a sharpened fused image, with some areas of the image having lost information, such as the branches of the tree on the right side and the branches of the small tree that is far away in the image, all of which are darker in the image, which is not conducive to obtaining information in a fully focused image; and the fusion image calculated by the proposed method is an all-focused image, with the details and edge information of the image being well preserved and with moderate brightness and ease of observation.

In Figure 7, we can denote that the fusion images calculated by the BGSC, GD, FusionDN, PMGI, and TEMST methods appear blurry, the images generated by BGSC and TEMST especially exhibiting severe distortion; the image computed by the NSCT method shows a basic full-focus result with some artifacts in the image, making it appear to have texture overlays; the U2Fusion method generates a dark image; and the fusion image calculated by the ZMFF method is very close to the result obtained by our algorithm. However, our fusion effect is better, with a clearer image and the almost seamless integration of all the information into the fully focused image.

In Figure 8, we can see that the fused images computed by the BGSC, FusionDN, and PMGI methods appear blurry, especially the forest information in the far distance of the images, making it difficult to observe the details; the images generated by the NSCT, TEMST, and ZMFF methods are similar; the fused image obtained by the GD method has high brightness and clarity; the fused image computed by the U2Fusion method has some dark regions, especially in the mouth area of the horse, with the loss of information being severe; and the fused image achieved by the proposed method has moderate brightness and retains more image information.

In Figure 9, we can see that the fusion images obtained by the BGSC, FusionDN, and PMGI approaches exhibit a certain degree of the blurring phenomenon, which makes it difficult to observe the information in the images, the image generated by the BGSC method especially not achieving full focus and the information on the left side of the image being severely lost; the NSCT, TEMST, and ZMFF methods all obtain a basic fully focused image from which information about the entire scene can be observed; the GD method gives a high-brightness fusion image but can also cause some details in the image to be lost; the fused image achieved by the U2Fusion method has many darker areas, causing severe information loss (for example, the information about the girl’s clothing and headscarf in the image cannot be captured correctly); the fusion image obtained by our algorithm fully preserves the information of the two source images. Additionally, it not only has moderate brightness, but the detailed information of the entire scene can also be fully observed and obtained.

### 4.2. Quantitative Comparisons 

**Figure 10:**
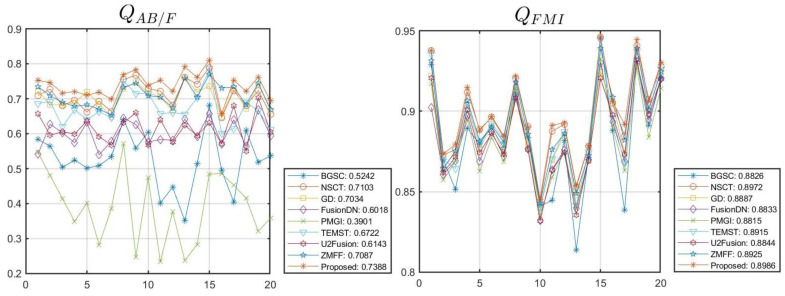

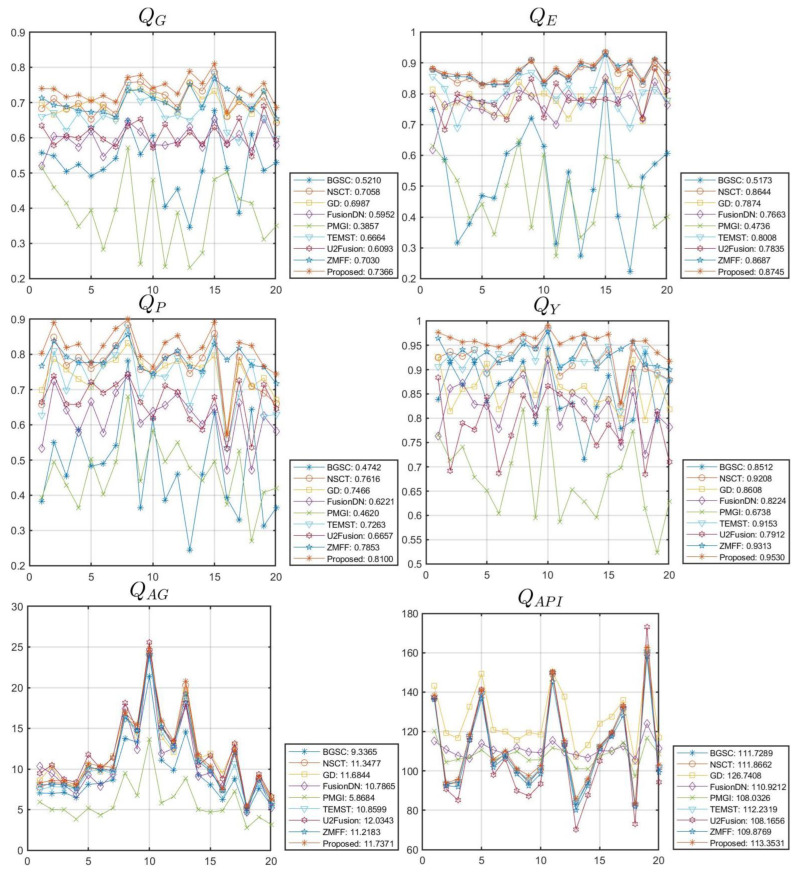
The line chart of metrics data.

In this section, the quantitative comparisons of different methods are shown in Table 1, Table 2, Table 3, Table 4, Table 5 and Table 6 and Figure 10. In Table 1, we can see that the metrics data $Q_{AB/F}$, $Q_{FMI}$, $Q_{G}$, $Q_{E}$, $Q_{P}$, $Q_{Y}$, and $Q_{AG}$ computed by the proposed method are the best; the metric data $Q_{API}$ generated by the GD method is the best, but the corresponding indicator result of our algorithm still ranks third. In Table 2, we can see that the metrics data *Q_AB/F_*, *Q_FMI_*, *Q_G_*, *Q_E_*, *Q_P_*, and *Q_Y_* generated by our method are the best. The metric data $Q_{AG}$ generated by the U2Fusion method is the best, while our method ranks second; the metric data $Q_{API}$ generated by the GD method is the best, and our method ranks fourth. In Table 3, we denoted that the metrics data *Q_AB/F_*, *Q_FMI_*, *Q_G_*, *Q_E_*, *Q_P_*, *Q_Y_* and *Q_AG_* computed by our method are the best; the metric data $Q_{API}$ generated by the FusionDN method is the best, and our method ranks fourth. In Table 4, we can see that the metrics data *Q_AB/F_*, *Q_FMI_*, *Q_G_*, *Q_E_*, *Q_P_*, and *Q_Y_* generated by our method are the best; the metrics data $Q_{AG}$ and $Q_{API}$ generated by the GD method are the best. In Table 5, we denoted that the metrics data $Q_{AB/F}$, $Q_{FMI}$, $Q_{G}$, $Q_{E}$, $Q_{P}$, $Q_{Y}$, and $Q_{AG}$ computed by our method are the best; the metric data $Q_{API}$ computed by the FusionDN method is the best.

Figure 10 shows the objective experimental results of different algorithms with respect to 20 sets of image pairs, and the data results of the same method with respect to different image pairs are connected into a curve, with the average indicator value on the right side of the indicator graph. Table 6 shows the average quantitative assessment of the different methods in Figure 10. In Figure 10 and Table 6, we can see that the average metrics data $Q_{AB/F}$, $Q_{FMI}$, $Q_{G}$, $Q_{E}$, $Q_{P}$, and $Q_{Y}$ generated by our method are the best; the average metrics date $Q_{AG}$ and $Q_{API}$ generated by the U2Fusion and GD methods are the best, respectively, and the two corresponding average metrics data computed by our method rank second. 

Through rigorous qualitative and quantitative evaluation and analysis, the results show that our algorithm stands out in the fusion effect of multi-focus images. Compared with state-of-the-art algorithms, we have achieved the best fusion effect and have the advantages of rich information and clear images.

## 5. Conclusions

In this paper, a novel multi-focus image fusion method based on the distance-weighted regional energy and structure tensor in NSCT domain was proposed. The structure tensor-based fusion rule was utilized to fuse low-frequency sub-bands, and the distance-weighted regional energy-based fusion rule was utilized to fused high-frequency sub-bands. The proposed method was experimented on the Lytro dataset with 20 paired images, and the fusion results demonstrate that our method can generate state-of-the-art fusion performance in terms of image information, definition, and brightness, making the seamless fusion of multi-focus images possible. In future work, we will improve and expand the application of this algorithm in the field of multi-modal image fusion so that it has better universality in image fusion.

## Figures and Tables

**Table 1:** The quantitative assessment of different methods in Figure 5.

	*Q_AB/F_*	*Q_MFI_*	*Q_G_*	*Q_E_*	*Q_P_*	*Q_Y_*	*Q_AG_*	*Q_API_*
BGSC	0.5083	0.8910	0.5104	0.4608	0.4902	0.8714	8.2490	103.9694
NSCT	0.6931	0.8963	0.6934	0.8322	0.7818	0.9205	10.1363	104.1275
GD	0.6843	0.8914	0.6874	0.7312	0.7718	0.8186	9.6352	120.8782
FusionDN	0.5398	0.8872	0.5459	0.7274	0.5769	0.7789	7.8779	110.6457
PMGI	0.2819	0.8835	0.2829	0.3429	0.4029	0.6042	4.3240	105.6984
TEMST	0.6632	0.8924	0.6633	0.7713	0.7702	0.9323	9.8586	104.3450
U2Fusion	0.5919	0.8863	0.5957	0.7646	0.6907	0.6872	10.0790	97.9331
ZMFF	0.6702	0.8898	0.6736	0.8290	0.7763	0.9146	9.8506	101.9007
Proposed	0.7190	0.8968	0.7193	0.8419	0.8245	0.9461	10.3594	105.8613

**Table 2:** The quantitative assessment of different methods in Figure 6.

	*Q_AB/F_*	*Q_MFI_*	*Q_G_*	*Q_E_*	*Q_P_*	*Q_Y_*	*Q_AG_*	*Q_API_*
BGSC	0.6042	0.8409	0.6059	0.6289	0.6181	0.9424	21.4182	100.9177
NSCT	0.7292	0.8431	0.7321	0.8345	0.7498	0.9861	24.1701	100.8183
GD	0.7172	0.8397	0.7211	0.8010	0.7403	0.9313	24.0096	118.4325
FusionDN	0.5762	0.8321	0.5800	0.7486	0.6380	0.9205	24.6566	109.2992
PMGI	0.4743	0.8352	0.4806	0.6013	0.5841	0.8205	13.6582	105.7407
TEMST	0.7159	0.8404	0.7193	0.8274	0.7421	0.9815	23.7422	101.4723
U2Fusion	0.5667	0.8320	0.5712	0.7219	0.6186	0.8666	25.5782	93.3662
ZMFF	0.7089	0.8429	0.7120	0.8316	0.7443	0.9772	24.0680	98.6039
Proposed	0.7381	0.8453	0.7412	0.8398	0.7649	0.9893	24.7951	102.7896

**Table 3:** The quantitative assessment of different methods in Figure 7.

	*Q_AB/F_*	*Q_MFI_*	*Q_G_*	*Q_E_*	*Q_P_*	*Q_Y_*	*Q_AG_*	*Q_API_*
BGSC	0.3510	0.8138	0.3463	0.2734	0.2438	0.7150	14.5754	82.8519
NSCT	0.7609	0.8528	0.7562	0.8874	0.7451	0.9556	20.2824	84.1720
GD	0.7597	0.8477	0.7543	0.7906	0.7720	0.8668	19.6094	107.0923
FusionDN	0.6414	0.8398	0.6312	0.7718	0.6440	0.8356	17.8845	108.0876
PMGI	0.2369	0.8391	0.2309	0.3342	0.4773	0.6285	8.8447	101.0823
TEMST	0.6563	0.8416	0.6491	0.7598	0.6544	0.9165	18.4142	83.8760
U2Fusion	0.6239	0.8354	0.6157	0.7789	0.6156	0.7982	18.1150	70.1179
ZMFF	0.7585	0.8490	0.7513	0.8963	0.7652	0.9671	19.2529	79.9660
Proposed	0.7909	0.8535	0.7881	0.9041	0.7919	0.9720	20.7733	85.8043

**Table 4:** The quantitative assessment of different methods in Figure 8.

	*Q_AB/F_*	*Q_MFI_*	*Q_G_*	*Q_E_*	*Q_P_*	*Q_Y_*	*Q_AG_*	*Q_API_*
BGSC	0.6808	0.9452	0.6766	0.8362	0.6360	0.8873	8.0699	112.4417
NSCT	0.7890	0.9460	0.7850	0.9335	0.8585	0.9412	9.9235	112.0279
GD	0.7360	0.9247	0.7328	0.8463	0.7966	0.8397	11.8692	124.0953
FusionDN	0.6570	0.9302	0.6529	0.8512	0.6466	0.8367	9.5025	110.2910
PMGI	0.4846	0.9326	0.4817	0.5948	0.4954	0.6824	4.6840	107.4757
TEMST	0.7850	0.9446	0.7830	0.9336	0.8464	0.9477	10.1213	112.5434
U2Fusion	0.6308	0.9205	0.6295	0.7828	0.6792	0.7869	11.6540	105.0806
ZMFF	0.7695	0.9390	0.7673	0.9270	0.8293	0.9289	9.7681	111.2188
Proposed	0.8106	0.9463	0.8102	0.9367	0.8903	0.9723	10.3106	112.7676

**Table 5:** The quantitative assessment of different methods in Figure 9.

	*Q_AB/F_*	*Q_MFI_*	*Q_G_*	*Q_E_*	*Q_P_*	*Q_Y_*	*Q_AG_*	*Q_API_*
BGSC	0.6100	0.9308	0.6117	0.5300	0.6441	0.9339	4.6552	82.9920
NSCT	0.6804	0.9410	0.6808	0.8308	0.7105	0.9020	5.1003	82.5546
GD	0.6710	0.9301	0.6678	0.7118	0.7076	0.7964	5.3423	104.4747
FusionDN	0.5641	0.9328	0.5611	0.7192	0.4708	0.7252	4.7077	105.7243
PMGI	0.4156	0.9272	0.4147	0.4973	0.2699	0.6139	2.7373	97.1696
TEMST	0.6899	0.9375	0.6902	0.8064	0.7799	0.9433	5.1428	82.9629
U2Fusion	0.5494	0.9316	0.5469	0.7190	0.5362	0.6846	5.3458	72.9192
ZMFF	0.6831	0.9390	0.6807	0.8368	0.7691	0.9100	5.1441	81.7770
Proposed	0.7209	0.9444	0.7210	0.8440	0.8238	0.9587	5.4338	83.2692

**Table 6:** The average quantitative assessment of different methods in Figure 10.

	*Q_AB/F_*	*Q_MFI_*	*Q_G_*	*Q_E_*	*Q_P_*	*Q_Y_*	*Q_AG_*	*Q_API_*
BGSC	0.5242	0.8826	0.5210	0.5173	0.4742	0.8512	9.3365	111.7289
NSCT	0.7103	0.8972	0.7058	0.8644	0.7616	0.9208	11.3477	111.8662
GD	0.7034	0.8887	0.6987	0.7874	0.7466	0.8608	11.6844	126.7408
FusionDN	0.6018	0.8833	0.5952	0.7663	0.6221	0.8224	10.7865	110.9212
PMGI	0.3901	0.8815	0.3857	0.4736	0.4620	0.6738	5.8684	108.0326
TEMST	0.6722	0.8915	0.6664	0.8008	0.7263	0.9153	10.8599	112.2319
U2Fusion	0.6143	0.8844	0.6093	0.7835	0.6657	0.7912	12.0343	108.1656
ZMFF	0.7087	0.8925	0.7030	0.8687	0.7853	0.9313	11.2183	109.8769
Proposed	0.7388	0.8986	0.7366	0.8745	0.8100	0.9530	11.7371	113.3531

## Data Availability

Not applicable.
